# Research on Pedestrian Indoor Positioning Based on Two-Step Robust Adaptive Cubature Kalman Filter with Smartphone MEMS Sensors

**DOI:** 10.3390/mi14061252

**Published:** 2023-06-14

**Authors:** Jijun Geng, Xuexiang Yu, Congcong Wu, Guoqing Zhang

**Affiliations:** 1 Coal Industry Engineering Research Center of Mining Area Environmental and Disaster Cooperative Monitoring, Anhui University of Science and Technology, Huainan 232001, China; xxyu@aust.edu.cn (X.Y.); 1993001@aust.edu.cn (G.Z.); 2 School of Geomatics, Anhui University of Science and Technology, Huainan 232001, China; 3 Anhui Provincial Key Laboratory of Joint Construction Disciplines for Urban Real Scene 3D and Intelligent Security Monitoring, Huainan 232001, China; 4 Key Laboratory of Aviation-Aerospace-Ground Cooperative Monitoring and Early Warning of Coal Mining-Induced Disasters of Anhui Higher Education Institutes, Anhui University of Science and Technology, KLAHEI (KLAHEI18015), Huainan 232001, China; 5 School of Graduate, Anhui University of Science and Technology, Huainan 232001, China; 2022212@aust.edu.cn

**Keywords:** MEMS sensor, indoor positioning, two-step RACKF, adaptive factor, robust factor

## Abstract

With the development of location-based service (LBS), indoor positioning based on pedestrian dead reckoning (PDR) has become a hot research topic. Smartphones are becoming more popular for indoor positioning. This paper proposes a two-step robust-adaptive-cubature Kalman filter (RACKF) algorithm based on smartphone micro-electro-mechanical-system (MEMS) sensor fusion for indoor positioning. To estimate pedestrian heading, a quaternion-based robust-adaptive-cubature Kalman filter algorithm is proposed. Firstly, the model noise parameters are adaptively corrected based on the fading-memory-weighting method and the limited-memory-weighting method. The memory window of the limited-memory-weighting algorithm is modified based on the characteristics of pedestrian walking. Secondly, an adaptive factor is constructed based on the partial state inconsistency to overcome filtering-model deviation and abnormal disturbances. Finally, to identify and control the measurement outliers, the robust factor based on maximum-likelihood estimation is introduced into the filtering to enhance the robustness of heading estimation and support more robust dynamic-position estimation. In addition, based on the accelerometer information, a nonlinear model is constructed and the empirical model is used to estimate the step length. Combining heading and step length, the two-step robust-adaptive-cubature Kalman filter is proposed to improve the pedestrian-dead-reckoning method, which enhances the adaptability and robustness of the algorithm and further improves the accuracy of the plane-position solution. The adaptive factor based on the prediction residual and the robust factor based on the maximum-likelihood estimation are introduced into the filter to improve the adaptability and robustness of the filter, reduce the positioning error, and improve the accuracy of the pedestrian-dead-reckoning method. Three different smartphones are used to validate the proposed algorithm in an indoor environment. Additionally, the experimental results confirm the algorithm’s effectiveness. From the results of the three smartphones, the root mean square error (RMSE) of the indoor-positioning results obtained by the proposed method is about 1.3–1.7 m.

## 1. Introduction

With the rapid development of mobile devices, location-based service (LBS) has become increasingly important [1]. There has been a dramatic increase in pedestrian demand for LBS both indoors and outdoors. Global Navigation Satellite System (GNSS) positioning and navigation technology, including GPS, GLONASS, Galileo, the Beidou navigation satellite system, and other regional systems, is reliable and accurate in outdoor space. However, GNSS positioning technology is not always available, especially in environments without satellite signals, such as indoor environments, underground environments, and urban-occlusion areas. Usually, it is common for people to spend most of their time indoors. Due to the limitation of satellite signals, GNSS-based solutions are ineffective in such scenarios, and in indoor environments, satellite signals are blocked by buildings, resulting in a large number of location blind spots and even an inability to receive GNSS signals. It is currently impossible for satellite positioning methods to meet the requirements of indoor positioning [2]. Consequently, indoor positioning requires additional positioning technology [3]. The complex layout of the indoor environment also poses various challenges to the positioning method, such as multipath propagation, non-line-of-sight conditions, signal attenuation, noise interference, etc. The construction of a stable, high-precision indoor-positioning system has therefore become a research hotspot. Although indoor positioning has become the focus of research in recent years, due to the complexity and variability of the actual indoor environment, excellent solutions have not been found. Over the past few decades, traditional indoor-positioning technology has relied on a variety of infrastructure support, including Wi-Fi, Bluetooth, ultra-wideband (UWB), radio-frequency identification, etc. These techniques are widely used for indoor positioning; however, the disadvantage of these methods is the need to build and maintain infrastructure [4]. Although the above methods are feasible in terms of positioning accuracy, the implementation of most existing positioning systems is based on infrastructure, which is usually difficult. Due to severe signal fluctuations caused by complex indoor radio-propagation conditions, Wi-Fi positioning cannot guarantee accurate positioning results. Besides, there are many places where there are no or few Wi-Fi APs, which makes localization difficult. Affected by the transmission distance of the Bluetooth signal, the coverage of Bluetooth positioning is limited. The cost of UWB is very high, and the signal-interference problem must be solved to coexist with the existing narrowband system. The RFID positioning system uses infrared beacons to find the user’s location. The limited infrared range limits the positioning of RFID in a wide range, and the use of RFID requires additional hardware-installation costs. Therefore, a practical indoor pedestrian-tracking method should consider the indoor environment instead of relying on a pre-trained database or floor plan, which is the key to developing an effective indoor-positioning system. The task of providing precise indoor positioning with low-cost devices is still difficult.

The micro-electro-mechanical-system (MEMS) positioning solution is more competitive than other methods because it does not rely on existing indoor infrastructure, which is particularly crucial for indoor positioning because other indoor-positioning technologies require additional installation or installation of specific sensors. MEMS sensors are small, lightweight, low cost, and independent. MEMS sensor devices are becoming increasingly popular and cheaper, and have been widely used in various fields and applications, such as motion monitoring and management, healthcare, location-based services (LBS), and navigation. In addition, the advancement of micro-electro-mechanical-system (MEMS) technology has made accelerometers, magnetometers and gyroscopes more and more accurate, lightweight, and low cost, which has greatly promoted their application in indoor positioning [5]. Accelerometers, magnetometers, and gyroscopes provide beaconless solutions. Due to the quick advancement of sensing technology, MEMS sensors composed of multiple sensors have attracted more and more attention. Various types of technologies have been introduced to achieve pedestrian positioning and navigation. Most smartphones currently support MEMS sensors [6]. Many studies have focused on using smartphone MEMS sensors for pedestrian dead reckoning (PDR) [7]. A PDR system based on accelerometers, magnetometers, and gyroscopes of smartphones can track the location of indoor users. MEMS positioning technology based on smartphones is usually used in PDR systems [6,7,8]. Therefore, the MEMS sensor data from a smartphone can be used to estimate the location information of the pedestrian conveniently. In addition, the PDR system on a smartphone is independent and does not require any external infrastructure, which can be used anywhere and at any time, with just a smartphone, without the need for a large infrastructure. As a result, compared to other positioning systems, the PDR method based on MEMS sensors is more practical.

The traditional dead-reckoning method uses inertial sensors, including accelerometers and gyroscopes, to estimate the relative position, and obtains the current position by adding an estimated displacement based on distance and heading data related to the previously estimated position [9]. However, the output of a gyroscope sensor is characterized by bias, bias instability, and other errors, which are integrated through the positioning and navigation equations to generate position errors that increase over time. To reduce the error caused by this integral drift, a feasible solution is to install the inertial sensor on the foot and estimate the position through the zero-velocity update algorithm [10]. When pedestrians walk, there is a standing stage. At this time, the speed of the foot is actually zero, which can avoid the acceleration-integration error. However, position accuracy is characterized by the accuracy of foot orientation. Once a constant heading error occurs, the position error increases linearly with time. The zero-velocity update-algorithm detection is also an important factor in step-length estimation. On the one hand, it must have a low error-detection rate, because the wrong steps will increase the position error. On the other hand, accurately estimating the start and end of each step helps to improve accuracy. PDR technology based on smartphone MEMS sensors is much more convenient than positioning technology based on inertial sensors. The PDR method is an economical and efficient choice, which is more suitable for pedestrian-gait patterns. A mobile phone is embedded with a variety of sensors, such as accelerometers, gyroscopes, and magnetometers, which can be used to construct PDR models. The PDR method provides an indoor-positioning method, including three aspects: heading estimation, stride detection, and step estimation [11]. The heading is the focus of this research. Due to the inaccurate estimation of heading and step length, the error of PDR positioning will increase with time. However, the PDR system can cause drift-error accumulation along walking distance, especially for cheap but noisy sensors of smartphones. Many studies have focused on PDR based on mobile-phone MEMS sensors. At present, some research on PDR has begun. However, most studies still lack comprehensive consideration. Each gyroscope and magnetometer can be used to infer the user’s heading, but both sensors have shortcomings in indoor positioning and navigation [3]. Due to the shortcomings of inertial sensors and magnetic sensors, a single type of sensor cannot provide accurate heading information. The heading calculated by the gyroscope is susceptible to drift problems and eventually accumulates errors over time, resulting in boundaryless directional-drift errors, because the measurement error accumulates when the data are fused. The magnetometer estimates the user’s heading by measuring the size of the earth’s magnetic field, but the indoor scene is susceptible to natural and magnetic-field sources, and the reading of the magnetometer is easily affected by the iron-containing material near the sensor [9]. Therefore, it needs to fuse data from different sensors to provide the best estimation of the heading. By combining gyroscope and magnetic sensors, numerous heading-estimate methods have been developed during the last few decades. The most commonly used methods are complementary filters (CF) and Kalman filters (KF) [7]. In the complementary-filter algorithm, data from the gyroscope are integrated to obtain the heading, and data from the accelerometer are used to estimate the gyroscope bias. However, it should be noted that they are all constant-gain complementary filters, and the estimation accuracy of this method depends on the accelerometer. Although the calculation cost of CF is low and the process is simple, the heading accuracy obtained by CF is lower than that of KF, and the result of CF in a dynamic environment is worse than that of the Kalman filter. The Kalman filter (KF) and extended Kalman filter (EKF) are the two most well-known and widely used methods, which have been widely used in various fields, especially in direction estimation [12]. However, it should be noted that the implementation of EKF causes linearization errors in the Kalman filter and increases the computational complexity [13]. To avoid the linearization process of the measurement model and reduce the computational load of the EKF, the unscented Kalman filter (UKF) and the cubature Kalman filter (CKF) are proposed [14]. Compared with the EKF, the CKF uses the cubature point set to approximate the mean and variance [15], avoiding the linearization of the nonlinear system. Additionally, the CKF is more adaptable and has stricter mathematical derivation than the UKF [15,16]. The CKF is a good method to deal with nonlinear estimation problems. Similar to the KF, the CKF can perform better when precise and comprehensive noise-distribution information is required [9]. Prior noise statistics, however, are frequently unknown or time-varying in real-world applications. Although many researchers have proposed robust-filtering algorithms and adaptive-filtering algorithms to solve these problems, there is still a lack of comprehensive evaluation [3,9,17]. Since the coordinates between the user and his/her device are inconsistent, different ways of carrying the device require different solutions to derive the user’s heading, which is relatively easy to determine by integrating the heading of the smartphone and the corresponding rotation matrix. [18,19,20]. Taking into account the algorithm adaptability and robustness, this paper proposes a two-step robust-adaptive-cubature Kalman filter (RACKF) for pedestrian indoor positioning. A robust-adaptive-cubature Kalman filter based on a quaternion is used to estimate pedestrian heading. Combining the fading-memory-weighting method and the limited-memory-weighting method, the noise parameters of the model are adjusted. An adaptive factor is constructed based on the partial-state inconsistency, which overcomes the influence of filtering-model error and abnormal disturbance. The robust factor based on maximum-likelihood estimation (M-estimator) is used to identify and control the measurement outliers. In addition, in this paper, the heading-angle-selection method of each step of PDR is improved, and the average value of one step-heading value is selected as the heading value of this step, which improves the reliability of the heading angle and reduces the randomness of the heading angle. Many studies have shown that the step length estimated by different methods is not much different [17]. In this paper, the nonlinear model is used to estimate the step length, and the maximum and minimum accelerations in one step are taken as the characteristic quantities. Combining the heading and step of pedestrians, this paper proposes a robust-adaptive-cubature Kalman filter to calculate the position information of pedestrians, further improve the positioning accuracy of pedestrians, and reduce the cumulative error of the heading angle. In general, the difference between the step length and heading of the previous step and the subsequent step is very small when the pedestrian walks normally, so the step length and the heading of the previous step can be regarded as a priori estimation of the step length and the heading of the subsequent step. The robust-adaptive-cubature Kalman filter is used to realize the PDR method. Many indoor-positioning systems assume the availability of the site map and use the site map to improve positioning accuracy by using prior knowledge of the path and wall in the building [21,22,23]. However, obtaining maps of anonymous buildings is not always possible, and we believe that any realistic, universal indoor-positioning system should be free from this fundamental limitation [9]. The proposed method can be easily used anywhere since the system does not require any anchor or physical-map information. Autonomous and independent pedestrian indoor positioning is the main goal of our research. The two-step robust-adaptive-cubature Kalman filter for indoor positioning proposed in this paper can be used anywhere and at any time and provides a lightweight positioning model. It can effectively reduce the influence of sensor cumulative error on position calculation and improve positioning accuracy. In general, the contributions of this paper can be summarized as follows.

First of all, by fusing the data of the magnetometer and the gyroscope sensor, a robust-adaptive-cubature Kalman filter based on a quaternion is proposed to optimize the heading estimation. The adaptive-correction-model system noise is combined with the fading-memory factor and the limited-memory-weighting method. An adaptive factor based on partial-state inconsistency is used to weaken the influence of filter-model error and abnormal disturbance. The robust factor based on maximum-likelihood estimation is used to identify and control the measurement outliers.

In addition, a robust-adaptive-cubature Kalman filter is proposed to improve the PDR method, which enhances the adaptability and robustness of the algorithm and further improves the accuracy of the plane-position solution. The adaptive factor based on the prediction residual and the robust factor based on the maximum-likelihood estimation are introduced into the filter to improve the adaptability and robustness of the filter, reduce the positioning error, and improve the accuracy of the PDR method.

The organization of this paper is as follows: The two-step robust-adaptive-cubature Kalman filter proposed in this paper is described in detail in Section 2. In Section 3, the experiments are explained and the results analysis is provided. In Section 4, the shortcomings of the experiment are discussed. Finally, the conclusions and future work are presented in Section 5.

## 2. Materials and Methods

**Figure 1:**
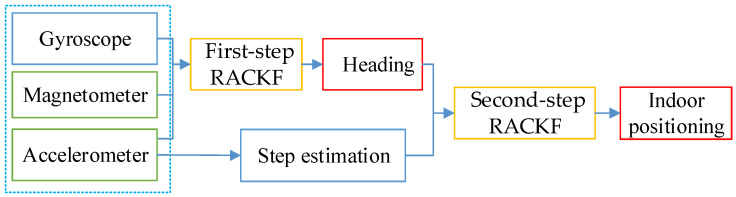
The framework of the two-step robust-adaptive-cubature Kalman method.

Low-cost MEMS sensors embedded in smartphones, such as accelerometers, magnetometers, and gyroscopes, provide raw data for heading estimation [17]. Although magnetometers can calculate the heading based on measured data in quasi-static conditions or magnetically clean environments, these values are easily affected by the surrounding environment or other factors, resulting in significant fluctuations around the true value [24,25,26]. In addition, although the heading may also be computed using angular velocity, the estimation is unreliable due to the accumulation of gyroscope-sensor errors during the integration process, especially during long-term operation [27,28,29]. Therefore, the robust-adaptive-cubature Kalman filtering algorithm is used to fuse the above two heading-estimation methods to obtain more accurate results. At the same time, a nonlinear step model is constructed with accelerometer information. Combining the estimated heading and step information, this paper proposes a robust-adaptive-cubature Kalman filter method to improve the PDR method. In the following, the process of the two-step robust-adaptive-cubature Kalman filter algorithm for indoor positioning is described in detail, as Figure 1.

### 2.1. Heading Estimation with the Quaternion

A quaternion is a parametric way to represent the heading. Because of the advantages of the small amount of calculation and global non-singularity, quaternions have been widely used. Quaternions:

(1) $$q\left(q_{0},q_{1},q_{2},q_{3}\right)=q_{0}+q_{1}i+q_{2}j+q_{3}k$$

where *q*_0_, *q*_1_, *q*_2_, and *q*_3_ are real numbers, and *i*, *j*, and *k* are unit vectors.

The heading can be calculated with the coordinate-transformation matrix from the b-coordinate system to the n-coordinate system. The transformation matrix can be described as:

(2) $$C_{b}^{n}=\left[\begin{array}{ccc} q_{0}^{2}+q_{1}^{2}-q_{2}^{2}-q_{3}^{2} & 2\left(q_{1}q_{2}-q_{0}q_{3}\right) & 2\left(q_{1}q_{3}+q_{0}q_{2}\right)\\ 2\left(q_{1}q_{2}+q_{0}q_{3}\right) & q_{0}^{2}-q_{1}^{2}+q_{2}^{2}-q_{3}^{2} & 2\left(q_{2}q_{3}-q_{0}q_{1}\right)\\ 2\left(q_{1}q_{3}-q_{0}q_{2}\right) & 2\left(q_{2}q_{3}+q_{0}q_{1}\right) & q_{0}^{2}-q_{1}^{2}-q_{2}^{2}+q_{3}^{2} \end{array}\right]$$

In addition, the axes of the navigation-coordinate system in this paper point to the east, north, and up. The matrix as the coordinate-transformation matrix from the n-coordinate system to the b-coordinate system is shown in the following:

(3) $$C_{n}^{b}=\left[\begin{array}{ccc} cos\varphi cos\psi +sin\varphi sin\psi sin\theta & -cos\varphi sin\psi +sin\varphi cos\psi sin\theta & -sin\varphi cos\theta \\ sin\psi cos\theta & cos\psi cos\theta & sin\theta \\ sin\varphi \;cos\psi -cos\varphi sin\psi sin\theta & -sin\varphi sin\psi -cos\varphi cos\psi sin\theta & cos\varphi cos\theta \end{array}\right]$$

where *ψ* is the yaw angle, *φ* is the pitch angle, and *θ* is the roll angle.

Combining Equations (2) and (3), the Euler angle can be expressed by a quaternion as follows [3,9]:

(4) $$\begin{array}{c} \theta =\arcsin\left[2\left(q_{2}q_{3}+q_{0}q_{1}\right)\right]\\ \varphi =\arctan\left[-\frac{2\left(q_{1}q_{3}-q_{0}q_{2}\right)}{1-q_{1}{}^{2}-q_{2}{}^{2}+q_{3}{}^{2}}\right]\\ \psi_{m}=\arctan\left[\frac{2\left(q_{1}q_{2}-q_{0}q_{3}\right)}{1-q_{1}{}^{2}+q_{2}{}^{2}-q_{3}{}^{2}}\right] \end{array}$$

The heading *ψ* can be obtained by the yaw:

(5) $$\psi =\psi_{m}+D$$

where *D* is the local declination angle.

### 2.2. Cubature Kalman Filter Algorithm

The core of the cubature Kalman filter is to estimate the statistical characteristics of random variables after nonlinear transformation based on the basic cubature points generated by spherical-radial-cubature rules. The cubature Kalman filter system and measurement equations can be expressed as follows:

(6) $$\begin{array}{l} X_{k}=f(X_{k-1})+w_{k-1}\\ z_{k}=h\left(X_{k}\right)+v_{k} \end{array}$$

where *X_k_* and *z_k_* are the system-state vector and the measurement vector of time k, respectively; *h*( ) is a known vector map; and *W_k_*_−1_ and *v_k_* are the process and measurement noise, respectively.

(1) Cubature Rule

The mean and variance can be used to represent a Gaussian distribution, using a Gaussian filter to complete the state-estimation task, in the following form:

(7) $$\begin{array}{l} {\hat{x}}_{k|k}={\hat{x}}_{k|k-1}+W_{k}(z_{k}-{\hat{z}}_{k})\\ P_{k|k}=P_{k|k-1}-W_{k}P_{zz,k|k-1}W_{k}{}^{T}\\ W_{k}=P_{xz,k|k-1}P_{zz,k|k-1}{}^{-1} \end{array}$$

where *ẋ_k|k_* and *P_k|k_* are the mean and variance of the probability distribution *p* (*x_k_|z_k_*), respectively. *P_k|k_*_−1_ is the state-prediction vector and its covariance at time k, and *P_zz,k|k_*_−1_ is the prediction measurement and covariance. *P_xz,k|k_*_−1_ is the predicted cross-covariance. *W_k_* is the Kalman gain. The multidimensional weighted integral is considered as follows:

(8) $$I\left(T\right)=\int_{D}T\left(x\right)w\left(x\right)d\left(x\right)$$

where T( ) is an arbitrary function, *D* ⊆ Rn is an integral domain, and *w*(*x*) ≥ 0 is a known weight.

Based on the spherical-radial-cubature rule, the cubature Kalman filter can be used to calculate the above equation:

(9) $$I\left(T\right)\approx \overset{m}{\underset{i=0}{\sum }}w_{i}T\left(\xi_{i}\right)$$

where *w_i_* = 1/m, *i* = 1, 2, m, and *m* = 2n. *ξ*i is the cubature point at the intersection of the unit sphere and its axis.

(2) Cubature Kalman Filter Algorithm Process

**Time Update**

Cholesky decomposition:

(10) $$P_{k-1|k-1}=S_{k-1|k-1}S_{k-1|k-1}^{T}$$

The cubature points X*_i, k|k_*_−1_ can be calculated as:

(11) $$X_{i,k-1|k-1}=S_{k-1|k-1}\xi_{i}+{\hat{x}}_{k-1|k-1}$$

where *ζ_i_* is the basic cubature points.

Evaluate the propagated cubature points:

(12) $$X_{i,k|k-1}^{*}=f\left(X_{i,k|k-1},u_{k-1}\right)$$

where *f*( ) is the known function, and *u_k_*_−1_ is the system noise.

The state prediction ${\hat{x}}_{k|k-1}$ and the covariance matrix of the state prediction *P_k|k_*_−1_ can be obtained as follows:

(13) $${\hat{x}}_{k|k-1}=\frac{1}{m}\overset{m}{\underset{i=1}{\sum }}X_{i,k|k-1}^{*}$$

(14) $$P_{k|k-1}=\frac{1}{m}\overset{m}{\underset{i=1}{\sum }}X_{i,k|k-1}^{*}X_{i,k|k-1}^{*T}-{\hat{x}}_{k|k-1}{\hat{x}}_{k|k-1}^{T}+Q_{k-1}$$

where *Q_k_*_−1_ is the system-noise covariance.

**Measurement Update**

Factorize:

(15) $$P_{k|k-1}=S_{k|k-1}S_{k|k-1}^{T}$$

The cubature points X*_i,k|k_*_−1_ can be evaluated:

(16) $$X_{i,k|k-1}=S_{k|k-1}\xi_{i}+{\hat{x}}_{k|k-1}$$

Then, the transmission of cubature points *Z_i,k|k_*_−1_ can be obtained:

(17) $$Z_{i,k|k-1}=h\left(X_{i,k|k-1},v_{k}\right)$$

where *h*( ) is the known function, and *v_k_* is the measurement noise.

Then, the measurement prediction ${\hat{z}}_{k|k-1}$ can be described as:

(18) $${\hat{z}}_{k|k-1}=\frac{1}{m}\overset{m}{\underset{I=1}{\sum }}Z_{i,k|k-1}$$

Combining Equations (17) and (18), the innovation-covariance matrix *P_zz,k|k_*_−1_ can be estimated:

(19) $$P_{zz,k|k-1}=\frac{1}{m}\overset{m}{\underset{I=1}{\sum }}Z_{i,k|k-1}Z_{i,k|k-1}^{T}-{\hat{z}}_{k|k-1}{\hat{z}}_{k|k-1}^{T}+R_{k}$$

where *R_k_* is the measurement-noise covariance.

The cross-covariance matrix *P_xz,k|k_*_−1_ can be calculated as:

(20) $$P_{xz,k|k-1}=\frac{1}{m}\overset{m}{\underset{I=1}{\sum }}X_{i,k|k-1}Z_{i,k|k-1}^{T}-{\hat{x}}_{k|k-1}z_{k|k-1}^{T}$$

Combining Equations (19) and (20), the Kalman gain *W_k_* can be described as:

(21) $$W_{k}=P_{xz,k|k-1}P_{zz,k|k-1}^{-1}$$

The state update and the corresponding error covariance can be written as:

(22) $${\hat{x}}_{k|k}={\hat{x}}_{k|k-1}+W_{k}\left(z_{k}-{\hat{z}}_{k|k-1}\right)$$

(23) $$P_{k|k}=P_{k|k-1}-W_{k}P_{zz,k|k-1}W_{k}^{-1}$$

### 2.3. First Step RACKF Algorithm

**Figure 2:**
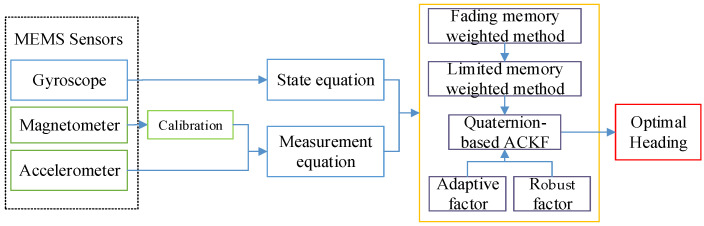
The frame of the first-step RACKF algorithm.

In terms of heading estimation, gyroscopes and magnetometers have certain limitations. The bias of the gyroscope increases with time, resulting in relative heading drift. Although magnetometers can calculate the heading based on the measured geomagnetic field in quasi-static conditions or magnetically clean environments, magnetometers are easily affected by the indoor environment. Therefore, the heading can be estimated by fusing these sensors, and the limitation of one sensor can be recovered by another sensor. In this paper, a robust-adaptive-cubature Kalman filter (RACKF) algorithm is proposed to fuse the data of MEMS sensors to obtain more accurate results. The fading-memory-weighting method and the limited-memory-weighting method are used to weigh the old data, and the model-noise parameters are adaptively corrected. Based on the characteristics of pedestrian walking, the memory window of the limited-memory-weighting algorithm is modified by using the latest step-length data of pedestrians. Based on the partial-state inconsistency, an adaptive factor is constructed to overcome the influence of filtering-model deviation and abnormal disturbance. To identify and control measurement outliers, a robust factor based on maximum-likelihood estimation is introduced in the filtering to enhance the robustness of heading estimation and support more robust dynamic-position estimation. The process of this method is explained as Figure 2:

#### 2.3.1. State Equation Based on Gyroscope

The quaternion q is a four-dimension vector and represents the changed heading from the previous quaternion, which can be calculated from:

(24) $$\dot{q}=\frac{1}{2}q⊗w$$

where w is the angular-rate vector.

The matrix form of Equation (24) is:

(25) $$\dot{q}=\frac{1}{2}M\left(w\right)q=\frac{1}{2}\left[\begin{array}{cccc} 0 & -w_{x} & -w_{y} & -w_{z}\\ w_{x} & 0 & w_{z} & -w_{y}\\ w_{y} & -w_{z} & 0 & w_{x}\\ w_{z} & w_{y} & -w_{x} & 0 \end{array}\right]\left[\begin{array}{c} q_{0}\\ q_{1}\\ q_{2}\\ q_{3} \end{array}\right]$$

where *w_x_*, *w_y_*, and *w_z_* are angular-rate values along the X, Y, and Z axes, respectively, of the device coordinate system.

The discrete form is [9]:

(26) $$q_{k+1}=[I*\cos(\vartheta /2)+A*dt*\sin(\vartheta /2)/\vartheta ]q_{k}$$

where *I* is the n × n unit matrix, *dt* is the sampling interval, and *A* is the incremental-angle matrix with its form of *w_x_*, *w_y_*, and *w_z_*.

#### 2.3.2. Measurement Equation Based on Accelerometer and Magnetometer

The conversion of the measured values u of the accelerometer and magnetometer and the quaternion q can be obtained from the relationship between the observation vectors in the body frame and the navigation frame as follows:

(27) $$u=\left[\begin{array}{l} a_{x}\\ a_{y}\\ a_{z}\\ m_{x}\\ m_{y}\\ m_{z} \end{array}\right]=\left(\begin{array}{c} 2(q_{1}q_{3}-q_{0}q_{2})\\ 2(q_{2}q_{3}+q_{0}q_{1})\\ q_{0}^{2}-q_{1}^{2}-q_{2}^{2}+q_{3}^{2}\\ 2(q_{1}q_{2}+q_{0}q_{3})m_{N}+2(q_{1}q_{3}-q_{0}q_{2})m_{U}\\ (q_{0}^{2}-q_{1}^{2}+q_{2}^{2}-q_{3}^{2})m_{N}+2(q_{2}q_{3}+q_{0}q_{1})m_{U}\\ 2(q_{2}q_{3}-q_{0}q_{1})m_{N}+(q_{0}^{2}-q_{1}^{2}-q_{2}^{2}+q_{3}^{2})m_{U} \end{array}\right)$$

where *a_x_*, *a_y_*, and *a_z_* represent the measurement of the accelerometer in the body-coordinate system. *m_x_*, *m_y_*, and *m_z_* represent the measurements of the magnetometer in the body-coordinate system. *m_N_* and *m_U_* stand for the components of a magnetic vector in the navigation-coordinate system.

In this paper, the magnetic-field-correction model is used to weaken the influence of hard iron and the scale factor as follows [9,17]:

(28) $$m=K\left(m^{*}+m_{0}\right)=diag\left(K_{x},K_{y},K_{z}\right)(\left[\begin{array}{c} m_{x}^{*}\\ \begin{array}{c} m_{y}^{*}\\ m_{z}^{*} \end{array} \end{array}\right]+\left[\begin{array}{c} m_{x0}\\ \begin{array}{c} m_{y0}\\ m_{z0} \end{array} \end{array}\right])$$

where *K* denotes a scale-transformation matrix. $m_{x}^{*}$, $m_{y}^{*}$, and $m_{z}^{*}$ are the raw measurements, and *m_x_*_0_, *m_y_*_0_, and *m_z_*_0_ are the biases.

Combining Equations (26) and (27), the process and observation models can be described as:

(29) $$\begin{array}{l} X_{k}=F_{k-1}X_{k-1}+w_{k-1}\\ z_{k}=h\left(X_{k}\right)+v_{k} \end{array}$$

where *w_k_*_−1_ and *v_k_* are the noise.

In the fading-memory-weighted method, combined with the Sage–Husa maximum-posterior-estimation algorithm and time-variant noise-statistic estimator, the state noise covariance ${\hat{Q}}_{k}$ can be expressed as follows [17]:

(30) $${\hat{Q}}_{k}=\left(1-d_{k}\right){\hat{Q}}_{k-1}+d_{k}[K_{k}\varepsilon_{k}\varepsilon_{k}^{T}K_{k}^{T}+P_{k|k}-(\frac{1}{2n}\overset{m}{\underset{i=1}{\sum }}X_{i,k|k-1}^{*}X_{i,k|k-1}^{*T}-{\hat{x}}_{k|k-1}{\hat{x}}_{k|k-1}^{T})]$$

where $d_{k}=(1-b)/(1-b^{k+1})$,b is the forgetting factor, $\varepsilon_{k}$ is the filter innovation, and $\varepsilon_{k}=z_{k}^{}-{\hat{z}}_{k|k-1}$.

Different from the traditional method, the nearest step data are selected as the length of the memory window in the pedestrian-walking process in this paper [9]. When the pedestrian is stationary, a sampling period is selected as the length of the memory window in the limited-memory-weighting method, and the weighting factor *β_i_* can be rewritten as:

(31) $$\beta_{i}=\beta_{i-1}b;0.95<b<0.99,\overset{k}{\underset{i=1}{\sum }}\beta_{i}=1$$

where $\beta_{i}=d_{w}b^{i-1}$, $d_{w}=\left(1-b\right)/\left(1-b^{w}\right)$, *b* is the forgetting factor.

The state noise covariance ${\hat{Q}}_{k}$ of the limited-memory adaptive filter can be expressed as [3]:

(32) $${\hat{Q}}_{k}=b{\hat{Q}}_{k-1}+d_{w}[K_{k}\varepsilon_{k}\varepsilon_{k}^{T}K_{k}^{T}+P_{k|k}-(\frac{1}{2n}\overset{2n}{\underset{i=1}{\sum }}X_{i,k|k-1}^{*}X_{i,k|k-1}^{*T}-{\hat{x}}_{k|k-1}{\hat{x}}_{k|k-1}^{T})]+d_{w}b^{w}{\hat{Q}}_{k-w}$$

where ${\hat{Q}}_{k-w}=W_{k-w}\varepsilon_{k-w}\varepsilon_{k-w}^{T}W_{k-w}^{T}+P_{k-w|k-w}-(\frac{1}{2n}\overset{2n}{\underset{i=1}{\sum }}X_{i,k-w|k-w-1}^{*}X_{i,k-w|k-w-1}^{*T}-{\hat{x}}_{k-w|k-w-1}{\hat{x}}_{k-w|k-w-1}^{T})$.

At the k-w moment, the state noise covariance of the restricted-memory-weighting method needs to be known. From the start time to the *k* − *w* time, the fading-memory-weighting method is used to calculate the state noise covariance. From the time *k* − *w* + 1, the state noise covariance is calculated by the limited-memory-weighting method. Combining the faded-memory-weighting method and the limited-memory-weighting method to estimate and correct the model-noise parameters, the accuracy of the filter estimation is improved.

The gain matrix *W_k_* in the first step of the robust-adaptive-cubature Kalman filter algorithm has different calculations. The complex motion state of pedestrians makes it difficult to establish an accurate function model. In addition, pedestrians are inevitably affected by abnormal external interference during the movement process, resulting in the state model not truly being able to reflect the movement of pedestrians. When the observation information is redundant, the state vector can be estimated directly by using the observation information. To overcome the influence of filter-model error and abnormal disturbance, an adaptive factor (α) based on partial-state inconsistency is used to overcome the abnormal influence of state disturbance. This paper uses a three-segment function to construct the adaptive factor with the statistic of the predicted-state discrepancy, which can be represented as:

(33) $$\partial_{k}=\{\begin{array}{c} 1,\;\;\;\;\;\;\;\;\;\;\;\;\;\;\;\;\;\;\;\;\;\;\;\;\;\;\;|\Delta {\tilde{X}}_{k}|\leq c0\\ \frac{c0}{\;|\Delta {\tilde{X}}_{k}|}(\frac{c1-\;|\Delta {\tilde{X}}_{k}|}{c1-c0}),\;c0<\;|\Delta {\tilde{X}}_{k}|\leq c1\\ 0,\;\;\;\;\;\;\;\;\;\;\;\;\;\;\;\;\;\;\;\;\;\;\;\;\;\;\;|\Delta {\tilde{X}}_{k}|>c1 \end{array}$$

where *c*0 and *c*1 are constants that can be tuned depending on the practical implementation, and $\Delta {\tilde{X}}_{k}$ is the statistic of the state-discrepancy statistic for judging the state-model errors.

(34) $$\Delta {\tilde{X}}_{k}={\left[\left\|{\tilde{X}}_{k}-X_{k|k-1}\right\|/tr\left({\hat{P}}_{k|k-1}\right)\right]}^{\frac{1}{2}}$$

where *tr*(·) stands for the trace of a matrix, and ${\tilde{X}}_{k}$ is a least-square estimator of the state.

The appropriate gain matrix *W_k_* is obtained as:

(35) $$W_{k}=(\frac{1}{m}\overset{m}{\underset{I=1}{\sum }}X_{i,k|k-1}Z_{i,k|k-1}^{T}-{\hat{x}}_{k|k-1}z_{k|k-1}^{T}){\left(\frac{1}{m}\overset{m}{\underset{I=1}{\sum }}Z_{i,k|k-1}Z_{i,k|k-1}^{T}-{\hat{z}}_{k|k-1}{\hat{z}}_{k|k-1}^{T}+\partial_{k}{\bar{R}}_{k}\right)}^{-1}$$

where $\partial_{k}$ is the adaptive factor, and ${\bar{R}}_{k}$ is the equivalent-weight matrix of the measurements.

To control the outliers in the measurements, the equivalent-weight matrix ${\bar{R}}_{k}$ of the measurements can be calculated as [30,31]:

(36) $${\bar{R}}_{k}=R_{k}/r_{i}$$

where *r_i_* is the variance-inflation factor, which is determined as follows [7]:

(37) $${\bar{r}}_{k_{ij}}=\{\begin{array}{c} 1\;\;\;\;\;\;\;\;\;\;\;\;\;\;\;\;\;\;\;|{\bar{v}}_{i}|\leq k_{0}\\ \frac{k_{0}}{\left|{\bar{v}}_{i}\right|}\cdot {\left(\frac{k_{1}-|{\bar{v}}_{i}|}{k_{1}-k_{0}}\right)}^{2},\;\;k_{0}<|{\bar{v}}_{i}|\leq k_{1}\;\\ 0\;\;\;\;\;\;\;\;\;\;\;\;\;\;\;\;\;\;\;|{\bar{v}}_{i}|>k_{1} \end{array}$$

where *k*_0_ and *k*_1_ are two thresholds, usually chosen as 1.5–3.0 and 3.0–8.0, respectively; and *v_i_* is the standardized residual.

### 2.4. Second Step RACKF Algorithm

**Figure 3:**
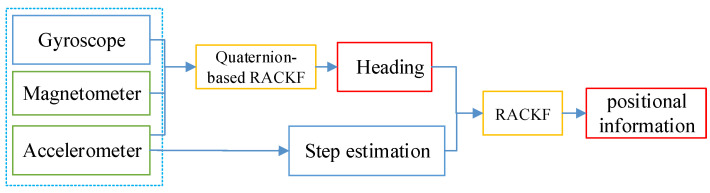
The framework of the second step of the RACKF algorithm.

At present, the study of PDR has received a lot of attention, but there is still a lack of comprehensive evaluation [3]. The PDR method on smartphones is independent and does not require any external infrastructure. This technology can be used anytime, anywhere, with just a smartphone and without the need for a huge infrastructure. Therefore, the smartphone-based PDR method will be the focus of future research. Due to inaccurate estimation of heading and step length, the error of PDR will increase over time, especially for smartphones with cheap and noisy built-in inertial sensors [17]. The traditional PDR method uses the heading and step length to calculate the plane position of the pedestrian, and the positioning accuracy depends on the estimation accuracy of the heading and step length. In this paper, a robust-adaptive-cubature Kalman filter is proposed to improve the PDR method, which enhances the adaptability and robustness of the algorithm and further improves the accuracy of the plane-position solution. This paper improves the method of selecting the heading angle of each step of the pedestrian. By selecting the average value of the heading in the step as the heading of the step, the fluctuation phenomenon starting from the peak (valley) time is weakened, the reliability of the heading angle is improved, and the randomness is weakened. A nonlinear model is constructed by using the information from the accelerometer to estimate the step length. Combining heading and step length, the second step of the robust-adaptive-cubature Kalman filter is proposed to improve PDR and estimate the position information of pedestrians. The adaptive factor based on the prediction residual and the robust factor based on the maximum-likelihood estimation are introduced into the filter to improve the adaptability and robustness of the filter, reduce the positioning error, and improve the accuracy of the dead-reckoning method. The process of this method is explained as Figure 3:

The speed estimation includes step length and frequency. In this paper, the adaptive step-length-estimation algorithm is used to take the maximum and minimum accelerations of pedestrians in one step as the characteristic quantity. The nonlinear-step-estimation model is as follows:

(38) $$L_{k}=K_{1}\left(a_{\max}-a_{\min}\right)+K_{2}\sqrt[{4}]{\left(a_{\max}-a_{\min}\right)}$$

where *L_k_* is the step length, and *a*_max_ and *a*_min_ are the maximum acceleration and minimum acceleration in one step, respectively. To improve the adaptability of the algorithm, two parameters, *K*_1_ and *K*_2_, can be adjusted in real time and added to the algorithm. These two parameters can be automatically adjusted according to the positioning result to adapt to the movement differences of each pedestrian.

At present, the gait-detection algorithm based on MEMS uses the peak-detection method to find the maximum acceleration of the fixed time window based on the periodic change in pedestrian acceleration. The main purpose of step-frequency detection is to identify the starting point of the stride from the continuous sensor data to facilitate data processing in the unit of the single step when calculating the subsequent step length and direction. In this paper, the method of “smooth window + peak detection + dynamic threshold” is used to detect cadence. The three-axis acceleration of the acceleration sensor and the rotation matrix are used to calculate the acceleration in the navigation-coordinate system. When performing pedestrian-gait detection, it is best to use the total acceleration in three directions during pedestrian walking. The numerical fluctuation of the total acceleration converted to a navigation system can reflect the human-walking law to a large extent. The total acceleration can be expressed as:

(39) $$a=\sqrt{a_{x}^{2}+a_{y}^{2}+a_{z}^{2}}$$

Then, based on the above equation, a smooth window with a length of 2n + 1 is used to filter the acceleration modulus. When the acceleration of human walking is removed from the gravity component of the earth, the acceleration value is positive or negative, and the acceleration value is zero. The zero-crossing method completes the judgment of pedestrian stride with this law. At the same time, the following conditions should be met: (1) The peak is greater than the threshold value, (2) the trough is less than the threshold value, (3) the difference between the peak and the trough is greater than the threshold value, and (4) the time difference between adjacent peaks is greater than the threshold value.

Usually, when a person walks normally, there is little difference in the step length and heading between the previous step and the next step [17]. Therefore, the step length and heading of the previous step can be used as a priori estimates of the next step length and heading. Combining the step length and heading, this paper proposes a robust-adaptive-cubature Kalman filter algorithm based on the PDR method to estimate pedestrian-position information, reducing the estimation error. In this paper, the state-transition equation and measurement equation are established as:

(40) $$\begin{array}{l} X_{k}=F_{k-1}X_{k-1}+w_{k-1}\\ z_{k}=H(X_{k})+v_{k} \end{array}$$

where $X_{k}={\left[\psi \;L\right]}^{T}$, $F_{k-1}=\left[\begin{array}{ll} 1 & 0\\ 0 & 1 \end{array}\right]$, $z_{k}=\left[\begin{array}{l} X\\ Y \end{array}\right]$, $h\left(u\right)=\left[\begin{array}{l} S*\cos(\varphi )\\ S*\sin(\varphi ) \end{array}\right]$, *w_k_*_−1_ and *v_k_* are the noises, ψ is the heading, and L is the step length.

In actual circumstances, it is difficult for pedestrians to maintain regular-motion mode [9]. Therefore, it is very difficult to establish an accurate function model. In addition, pedestrians are disturbed by the outside world during the movement, which makes the state model unable to reflect the real-motion law. Since the observation information is not redundant, the adaptive factor is constructed by using the prediction residual to overcome the filter-model error and the influence of abnormal disturbance [17]. In this paper, the adaptive factor is a two-stage function, which is a statistic of the predicted state difference and can be expressed as [3]:

(41) $$\partial_{k}=\{\begin{array}{c} 1,\;\;\;\;\;\;\;\Delta {\tilde{X}}_{k}\leq c0\\ \frac{c0}{\Delta {\tilde{X}}_{k}},\;\;\;\;\Delta {\tilde{X}}_{k}>c0 \end{array}$$

where *c*0 is a constant, which can be tuned depending on the practical implementation, and $\Delta {\tilde{V}}_{k}$ is the statistic of the predicted state discrepancy, defined as $\Delta {\tilde{X}}_{k}={\left[\left\|{\tilde{X}}_{k}-{\bar{X}}_{k}\right\|/tr\left(\mathrm{cov}(\Delta \tilde{V},\Delta {\tilde{V}}^{T}\right)\right]}^{\frac{1}{2}}$, where *tr*(·) stands for the trace of a matrix and ${\tilde{X}}_{k}$ is a least-square estimator of the state.

The adaptive factor is used to correct the innovation-covariance matrix to control the influence of the dynamic-model error. Having weakened the negative impacts of measurement outliers and state-model errors, the innovation-covariance matrix $P_{{}_{zz,k|k-1}}^{*}$ can be expressed as [3]:

(42) $$P_{zz,k|k-1}^{*}=\frac{1}{m}\overset{m}{\underset{I=1}{\sum }}Z_{i,k|k-1}Z_{i,k|k-1}^{T}-{\hat{z}}_{k|k-1}{\hat{z}}_{k|k-1}^{T}+\partial_{k}{\bar{R}}_{k}$$

where $\partial_{k}$ is the adaptive factor, and ${\bar{R}}_{k}$ is the equivalent-weight matrix of the measurements.

In this paper, the robust estimation based on maximum-likelihood estimation is used to correct the equivalent-weight matrix of the measurement to control the outliers in the measurement. Then, the diagonal ${\bar{r}}_{k_{ij}}$ and non-diagonal ${\bar{r}}_{k_{ij}}$ elements of the equivalent-weight matrix are determined as follows [17]:

(43) $${\bar{r}}_{k_{ii}}=\{\begin{array}{c} \frac{1}{\sigma_{ii}},\;\;\;\;\;\;\;\left|r_{k_{i}}^{\prime }\right|\leq c\\ \frac{c}{\left|r_{k_{i}}^{\prime }\right|}\cdot \frac{1}{\sigma_{ii}},\;\left|r_{k_{i}}^{\prime }\right|>c \end{array}$$

(44) $${\bar{r}}_{k_{ij}}=\{\begin{array}{c} \frac{1}{\sigma_{ij}},\;\;\;\;\;\;\;\;\;\;\;\;\;\;\;\;\;\;\;\;\left|r_{k_{i}}^{\prime }\right|\leq c\;and\;\left|r_{k_{j}}^{\prime }\right|\leq c\\ \frac{c}{\max\left\{\left|r_{k_{i}}^{\prime }\right|,\left|r_{k_{j}}^{\prime }\right|\right\}}\cdot \frac{1}{\sigma_{i,j}},\;\;\left|r_{k_{i}}^{\prime }\right|>c\;or\;\left|r_{k_{j}}^{\prime }\right|>c \end{array}$$

where $\sigma_{ii}$ and $\sigma_{ij}$ are diagonal and non-diagonal elements of the measurement-noise covariance matrix **R***_k_*, *c* is a constant, and $r_{k_{i}}^{\prime }$ denotes the standard residual and is calculated by:

(45) $$\left|r_{k_{i}}^{\prime }\right|=\left|\frac{r_{k_{i}}}{\sigma_{r_{k_{i}}}}\right|$$

## 3. Experiments and Results Analysis

### 3.1. Experimental-Site Layout

**Figure 4:**
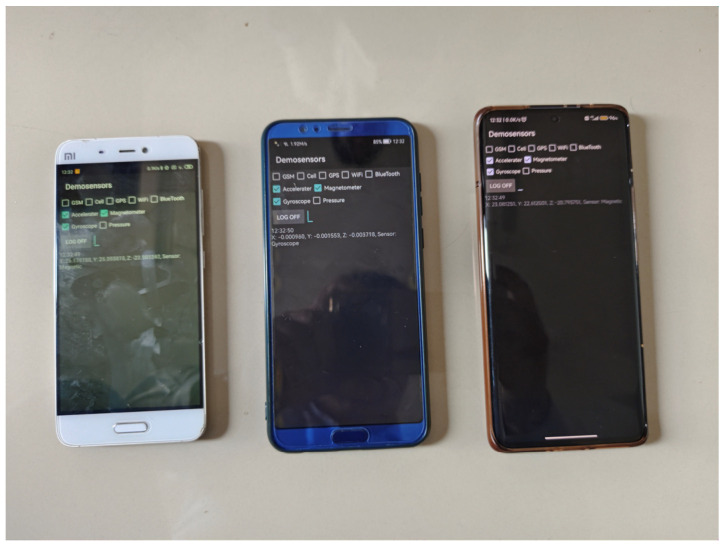
Three pieces of experimental equipment.

**Figure 5:**
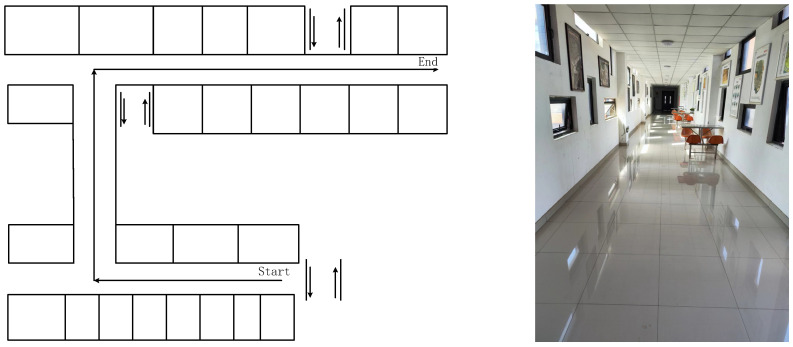
Floor plan of the site for the test.

To evaluate the proposed method, experiments were carried out in indoor scenes. Smartphones Xiaomi 5 (Mi 5), HONOR V10, and Xiaomi 12(Mi 12 Pro) were selected as the test equipment, as shown in Figure 4. Based on the data-acquisition software (2.0_ alpha) we developed, the sampling frequency of the data was 50 Hz. In addition, the experimental results of the three pieces of experimental equipment can be compared with each other to improve the reliability of the experimental results. In addition, since the sensors of each device are different, the experimental results of the three devices can increase the versatility of the proposed method. The initial-state noise and measurement-noise covariance matrix of the filter were determined by the experience of each measured-value output by the smartphone in the test [9]. To verify the accuracy of the proposed method, experiments were carried out on the fourth floor of a research building. To ensure the detection of pedestrian-positioning accuracy, we only considered the case where the user holds a smartphone, which is the most common pedestrian navigation mode. During the experiment, participants started from the starting point and reached the end point at a constant speed along the corridor, as shown in Figure 5. In indoor experiments, pedestrians held the device and kept it level. The trajectory of their walking position was 103.72 m. Participants maintained a uniform walking speed in the experiment. During the experiment, pedestrians held the smartphone in their hands. The x-axis of the smartphone was along the forward direction, the y-axis was perpendicular to the x-axis and the right, and the z-axis was perpendicular to the x–y plane. The experiment was completed by two people. The first person carried the experimental equipment and walked at a constant speed. The second participant used a mobile phone to photograph the position of each step of the previous person’s experimental process and obtained the real position of each step by pre-measuring the total length of the route.

### 3.2. Results Analysis

**Figure 6:**
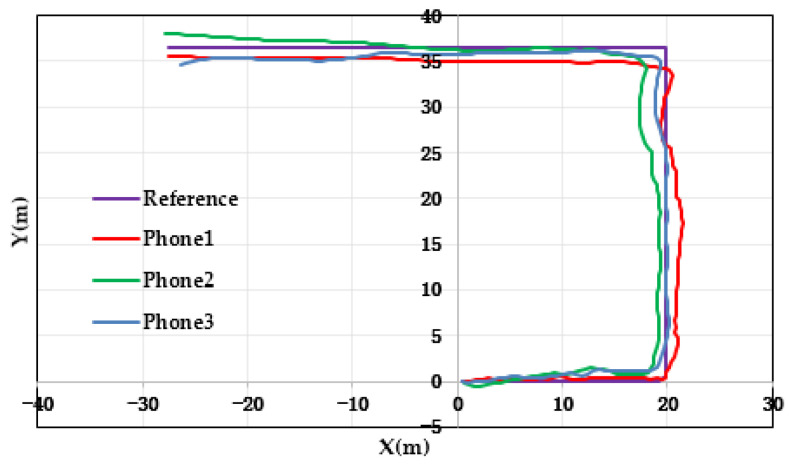
Distributions of location with respect to the three pieces of experimental equipment.

**Figure 7:**
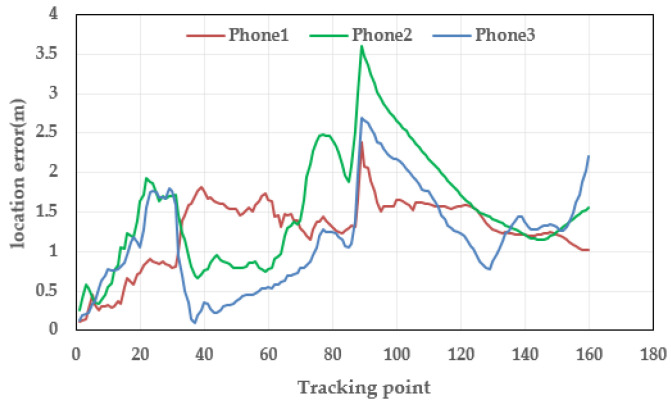
Distributions of location errors with respect to the three smartphones.

Figure 6 and Figure 7 show the results and errors of position tracking, which can reflect the position coordinates of the experiment. The purple line in Figure 6 is the reference trajectory, which was obtained from the field-measurement coordinates. The red line in the image is the result of positioning by the Xiaomi 5 mobile phone, the green line is the result of positioning by the HONOR V10 mobile phone, and the blue line is the result of positioning by the Xiaomi 12 Pro mobile phone. In Figure 6, it can be concluded that the positioning results of the three mobile phones were close to the reference trajectory. This is because the algorithm proposed in this paper introduces adaptive and robust factors, which reduce the accumulation of errors and improve the accuracy of position estimation. Figure 7 shows the positioning error of the three mobile phones. From Figure 7, it can be seen that the positioning error of the three mobile phones was not much different and the positioning accuracy was very high. In addition, the maximum error was in the corner. There were two corners in this experiment, and the error was relatively large at the two corners. This shows that the proposed algorithm needs to further improve the positioning accuracy of pedestrians in the corner.

**Figure 8:**
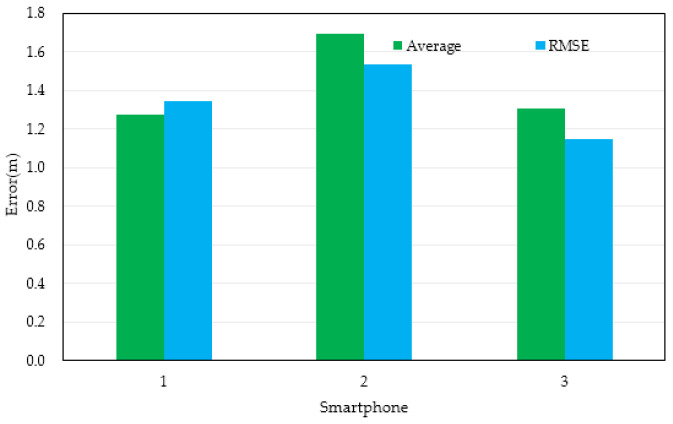
Distributions of the error of location results in location tracking for the three smartphones.

Table 1 and Figure 8 show the statistical data of position errors. Based on the positioning results of the three mobile phones in the table, it can be seen that the root mean square error (RMSE) of the positioning results of the proposed method was 1.3452, 1.5372, and 1.1508, and the average error of the positioning results was 1.2756, 1.6969, and 1.3086, respectively.

### 3.3. The Second Experiment and Results Analysis

**Figure 9:**
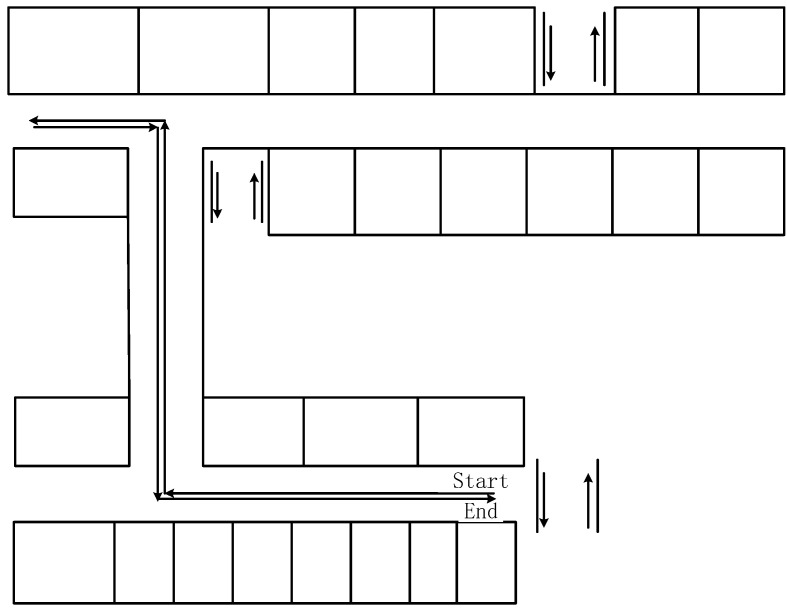
Floor plan of the site for the second test.

To further verify the superiority of the proposed method, the second test was conducted in the corridors on the fourth floor of a research building. The floor plan is presented in Figure 9. In the test, the smartphone was held at a constant speed and started from the starting point and returned to the start point along the corridor. The trajectory of the walking position was 128.24 m.

**Figure 10:**
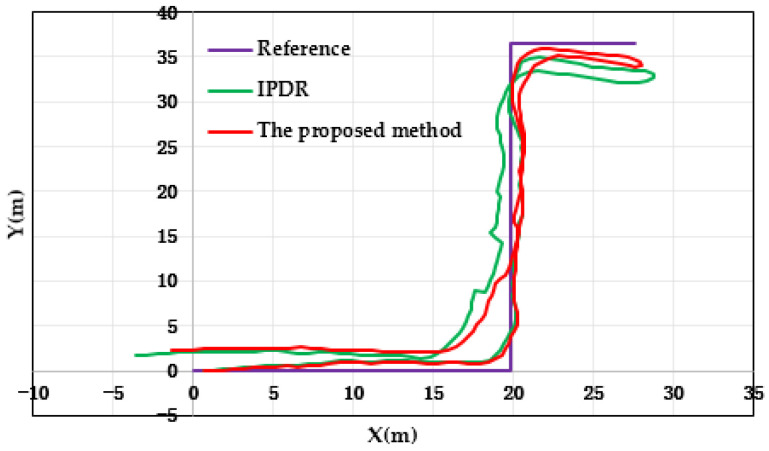
Distributions of location with respect to the two methods.

**Figure 11:**
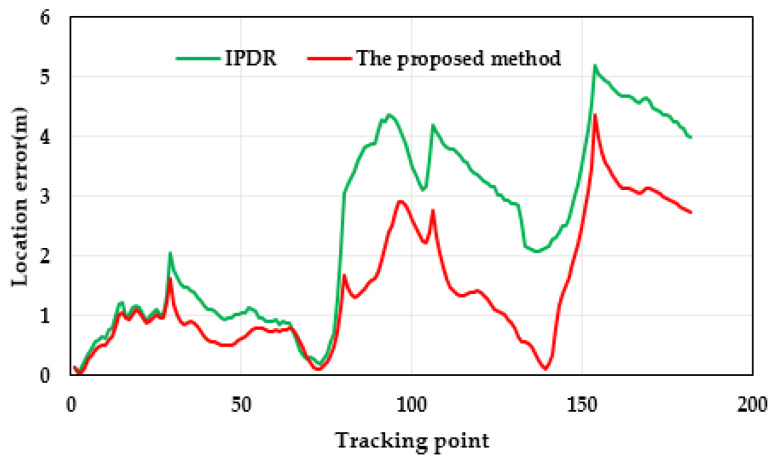
Distributions of location errors with respect to the two methods.

Figure 10 and Figure 11 show the results and errors of the position tracking, which reflect the position coordinates of the experiment. In Figure 10, the purple line is the reference trajectory, which was obtained from the field-measurement coordinates. The red line in the image is the result of positioning with the proposed method. The green line is the result of positioning with the IPDR method. From Figure 10, it can be concluded that the proposed method provided more stable and accurate location information compared with the IPDR algorithm. This is because the IPDR algorithm uses a quaternion-based robust-adaptive-cubature Kalman filter algorithm to estimate pedestrian heading and calculate pedestrian-position information with the PDR method. The proposed method uses a two-step robust-adaptive-cubature Kalman filter algorithm to calculate pedestrian-position information. Compared to the IPDR algorithm, the proposed method uses a robust-adaptive-cubature Kalman filter to reduce the positioning error and improve the accuracy of the IPDR method, which enhances the adaptability and robustness of the algorithm and further improves the accuracy of the plane-position solution. Figure 11 shows the positioning error of two methods, and it can be seen that the positioning error of the proposed method was smaller than that of the IPDR method. In addition, Table 2 shows the statistical data of the position errors for the two methods. Based on the positioning results in the table, it can be seen that the root mean square error (RMSE) and the average error of the positioning results of the proposed method were smaller than those of the IPDR method. Compared with the IPDR method, the RMSE and the average error of the proposed method diminished to about 38.93% and 41.54%, respectively.

In general, the above results show that the proposed two-step robust-adaptive-cubature Kalman filter method can not only provide the optimal model for heading estimation but also further optimize the IPDR algorithm. It is worth noting that based on the different smartphone experiments, it can be inferred that the proposed two-step robust-adaptive-cubature Kalman filter algorithm has the advantages of small error and high stability in indoor scenes. Therefore, it can be concluded that the proposed two-step robust-adaptive-cubature Kalman filter algorithm can obtain good positioning accuracy, making it more suitable for the application of low-cost MEMS sensors for pedestrian indoor positioning.

## 4. Discussion

In this paper, a two-step robust-adaptive-cubature Kalman filter based on the MEMS sensor of a smartphone is proposed for indoor pedestrian positioning. Although the first step of the robust-adaptive-cubature Kalman filter and the second step of the robust-adaptive-cubature Kalman filter in the two-step robust-adaptive-cubature Kalman filter proposed in this paper adopt adaptive factors and robust factors, they are different. The differences are as follows:

Firstly, only the first step of the robust-adaptive-cubature Kalman filter uses the fading-memory-weighting method and the limited-memory-weighting method to adaptively correct the statistical characteristics of the nonlinear system and reduce the estimation bias of the filter.

Secondly, the adaptive filtering of the two-step robust-adaptive-cubature Kalman filter has different value ranges for the adaptive factor. The three-stage function-adaptive factor is suitable only for the weighted average solution, not for the recursive solution. The two-stage function-form adaptive factor can be applied to the weighted average solution and the recursive solution. When the observation information is sufficient, the adaptive factor should adopt the three-stage function and the corresponding state-inconsistency statistics. When the observation information is seriously insufficient, the adaptive factor must be constructed based on the prediction residual and cannot be zero, and the non-zero adaptive factor must be used.

Finally, although the first step of the robust-adaptive-cubature Kalman filter and the second step of the robust-adaptive-cubature Kalman filter both use the robust factor based on the maximum-likelihood estimation, the two robust factors are different methods.

In addition, in the two-step robust-adaptive-cubature Kalman filter proposed in this paper, the first step of the robust-adaptive-cubature Kalman filter is to fuse the heading results calculated by the gyroscope and magnetic field, which further optimizes the heading estimation and improves the estimation accuracy. The second step of the robust-adaptive-cubature Kalman filter is to improve the traditional PDR method. The traditional PDR method uses the heading and step length to calculate the plane position of the pedestrian, and the positioning accuracy depends on the estimation accuracy of the heading and step length. In this paper, the second step of the robust-adaptive-cubature Kalman filter is used to improve the PDR method, which enhances the adaptability and robustness of the algorithm and further improves the accuracy of the plane-position solution.

Although the proposed method achieved stable and accurate positioning results, there are still some problems to be discussed.

(1) Different from most pedestrian-indoor-positioning methods, the method proposed in this paper is based on smartphone MEMS sensors to determine indoor positioning. Considering the adaptability and robustness of the filter, a two-step RACKF algorithm is proposed to estimate the pedestrian position to improve the positioning accuracy and weaken the error accumulation of the PDR method. However, the proposed method only applies to pedestrians holding a smartphone with their hands and maintaining it level. It does not apply to pocket and swing modes of the phone, which will be the subject of future research work. Based on these limitations, our future work will focus on a more comprehensive positioning model. In addition, because the two-step RACKF algorithm comprehensively considers robustness and adaptability, the complexity of the algorithm is increased.
(2) Due to the complex and changeable indoor environment, the influencing factors of pedestrian positioning are uncertain. Although the proposed two-step robust-adaptive-cubature Kalman filter indoor-positioning method can reduce the positioning-accumulation error of PDR to a certain extent, pedestrian-indoor-positioning error may still accumulate. Therefore, it is necessary to further improve the performance of the algorithm and reduce error accumulation.
(3) It can be inferred from the experimental results that the method proposed in this paper can meet the needs of most ordinary pedestrians. However, the error is relatively large in the place of turning. How to further improve the positioning accuracy of pedestrian turning is a future research direction.
(4) Pedestrian-behavior patterns in complex environments are complex and changeable. In actual scenes, pedestrian-walking patterns are changeable during walking, which may affect the accuracy of pedestrian positioning. It is necessary to conduct an in-depth analysis of multi-sensor data characteristics of smartphones and use recognition algorithms to intelligently identify pedestrian-behavior characteristics.

## 5. Conclusions

In this paper, a two-step robust-adaptive-cubature Kalman filter positioning method based on a smartphone MEMS sensor is proposed for pedestrian indoor positioning. The quaternion-based robust-adaptive-cubature Kalman filter (RACKF) algorithm is used to estimate the heading, which reduces the weight of the old data and adaptively modifies the model-noise parameters. The fading-memory-weighting method and the limited-memory-weighting method are combined to adaptively correct the statistical characteristics of the nonlinear system and reduce the estimation bias of the filter. An adaptive factor is constructed based on the partial-state inconsistency to overcome the influence of the filter-model error and abnormal disturbance. In addition, the robust factor based on maximum-likelihood estimation is used to identify and control the measurement outliers to enhance the robustness of heading estimation. Each step of the pedestrian contains multiple heading angles at different times, and the average heading of the step is selected as the heading of the step to reduce the influence of fluctuation. Step-length estimation is achieved by using accelerometer data in smartphones. In addition, based on PDR, a second step of the robust-adaptive-cubature Kalman filter is proposed to estimate pedestrian-position information. Combining the adaptive factor based on the prediction residual and the robust factor based on maximum-likelihood estimation, the adaptability and robustness of the filter are improved and the positioning error is reduced. The proposed indoor-positioning method effectively reduces the influence of sensor cumulative error on position calculation and improves positioning accuracy.

The experiment is carried out in an indoor environment to verify the superiority of the proposed method. The experimental results show that the two-step robust-adaptive-cubature Kalman filter can improve the indoor-positioning accuracy of pedestrians, and the algorithm can provide more stable and accurate position-estimation information. Therefore, the experimental results show that the proposed indoor-positioning method can provide an optimization model for pedestrian indoor positioning and navigation estimation. Therefore, it can be concluded that the proposed method can obtain better accuracy and make it more suitable for indoor positioning using low-cost MEMS sensors of smartphones.

In the future, we will focus on pedestrian-motion-pattern recognition to improve the accuracy of positioning. In addition, different ways of carrying mobile phones will also be the focus of our research.

## Figures and Tables

**Table 1:** Statistical results of pedestrian-location error (m).

Experimental Devices	Average	RMSE
Mi 5	1.2756	1.3452
HONOR V10	1.6969	1.5372
Mi 12 Pro	1.3086	1.1508

**Table 2:** Statistical results of pedestrian-location error (m) of second test.

Experimental Devices	Average	RMSE
IPDR	2.4726	2.9017
The proposed method	1.4456	1.7721

## Data Availability

The data presented in this study are available from the corresponding author on reasonable request.

## Abbreviations

LBS	location-based services
MEMS	micro-electro-mechanical systems
PDR	pedestrian dead reckoning
RACKF	Robust-adaptive-cubature Kalman filter
RMSE	root mean square error
GNSS	Global Navigation Satellite System
CF	complementary filter
KF	Kalman filter
EKF	extended Kalman filter
UKF	unscented Kalman filter
CKF	cubature Kalman filter
